# Challenges and advantages of being a scientific journal editor in the era of ChatGPT

**DOI:** 10.5935/0004-2749.2023-1003

**Published:** 2023

**Authors:** Rodrigo Pessoa Cavalcanti Lira, Eduardo Melani Rocha, Newton Kara-Junior, Dácio Carvalho Costa, Fernando Procianoy, Jayter Silva de Paula, Carolina P. B. Gracitelli, Tiago da Silva Prata, Caio V. Regatieri, Laurentino Biccas Neto, Monica Alves

**Affiliations:** 1 Department of Surgery, Medical School, Universidade Federal de Pernambuco, Recife, PE, Brazil; 2 Department of Ophthalmology, Otorhinolaryngology and Head & Neck Surgery, Ribeirao Preto Medical School, Universidade de São Paulo, Ribeirão Preto, SP, Brazil; 3 Department of Ophthalmology, Medical School, Universidade de São Paulo, São Paulo, SP, Brazil; 4 Department of Ophthalmology, Public Health School of Ceara, Universidade Estadual do Ceará, Fortaleza, CE, Brazil; 5 Department of Ophthalmology, Medical School, Universidade Federal do Rio Grande do Sul, Proto Alegre, RS, Brazil; 6 Department of Ophthalmology and Visual Science, Universidade Federal de São Paulo, São Paulo, SP, Brazil; 7 Ocular Oftalmologia. Vitoria, ES, Brazil; 8 Department of Ophthalmology & Otorhinolaryngology, Medical Sciences Faculty, Universidade Estadual de Campinas, Campinas, SP, Brazil

*Being a science journal editor is a challenging but rewarding profession that offers many benefits and opportunities for personal and professional growth. In this editorial, we will discuss some challenges and advantages of being a science journal editor.*

*The job of a scientific journal editor is far from easy, as they face a plethora of challenges that can make their work incredibly difficult. One of the primary challenges that scientific journal editors face is managing the peer--review process. Peer review is the cornerstone of scientific publishing, ensuring that the research published in journals is of high quality and rigorously scrutinized. However, managing the peer-review process can be time--consuming and complex, involving the coordination and finding reviewers, authors, and editors with high--quality. Moreover, editors need to ensure that the peer review process is fair and unbiased, which requires a deep understanding of the subject matter and a willingness to make tough decisions.*

*Another significant challenge that scientific journal editors face is staying up-to-date with the latest research and developments in their field. Editors need to be able to recognize the most significant research trends and topics in their field, as well as identify emerging areas of research that may be of interest to their readership. This requires a lot of reading and research, as well as attending conferences and networking with other researchers.*

*In addition to managing the peer-review process and staying up-to-date with the latest research, scientific journal editors also need to ensure that their journals are financially viable. This means balancing the need to publish high-quality research with the need to attract subscribers and generate revenue. Editors need to be able to identify topics and research that are likely to be of interest to their readership, as well as develop marketing strategies to attract new subscribers and retain existing ones.*

*Another demanding dare that scientific journal editors face is dealing with the pressure to publish. In today’s highly competitive academic environment, researchers are under increasing pressure to publish their research in high-impact journals. This can put pressure on editors to publish research that may not be of the highest quality or rigor, simply to meet their publishing quotas. Editors need to be able to resist this pressure and maintain their commitment to publishing only the highest quality research.*

*Finally, scientific journal editors also face the challenge of navigating the rapidly evolving landscape of scientific publishing. With the rise of open-access publishing, preprint servers, and other emerging technologies, editors need to be able to adapt to new publishing models and technologies while maintaining their commitment to high-quality research and rigorous peer review.*

*However, being a science journal editor is a highly rewarding profession that offers many benefits. One of the most significant benefits of being a science journal editor is the opportunity to shape the direction of scientific research. Editors have the power to select and publish research that is most likely to make a significant impact in their field. This means that editors can influence the development of new research topics and steer the field towards new and exciting areas of discovery.*

*Another benefit of being a science journal editor is the opportunity to work with some of the world’s leading researchers and experts. Editors have the chance to network with prominent scientists, attend conferences and workshops, and collaborate on research projects. This exposure to the latest research and emerging trends can provide editors with a unique perspective on the state of their field.*

*Being a science journal editor also offers the chance to make a significant contribution to scientific knowledge. By publishing high-quality research and ensuring that it is disseminated to the wider scientific community, editors play a critical role in advancing the field of science. This can be incredibly fulfilling for editors who are passionate about their work and the impact it can have on the world.*

*In addition to the intellectual and professional benefits, being a science journal editor can also be financially rewarding. Many scientific journals offer competitive salaries and benefits packages, as well as opportunities for career advancement. This can make it an attractive profession for those who are looking for a challenging and financially stable career.*

*Finally, being a science journal editor can be incredibly satisfying on a personal level. Editors have the chance to work with a team of professionals who are passionate about their work and committed to advancing scientific knowledge. This can create a supportive and collaborative work environment that can be incredibly rewarding for editors.*

*In conclusion, being a science journal editor offers many challenges and advantages. While the profession can be demanding and require significant effort and dedication, it also offers the opportunity to make a significant contribution to scientific knowledge and advance the field of science. For those who are passionate about advancing scientific knowledge and committed to the pursuit of excellence, being a science journal editor can be a rewarding and fulfilling career*^(1)^.

## Human authors’ contribution

The text presented in italic, except the title, was enti-rely written with the assistance of ChatGPT. This chat--generative pretrained transformer (ChatGPT), launched in November 2022, has authored 05 articles in the remainder of 2022 and 117 articles so far in 2023^(2)^.

ChatGPT was requested to produce a text about the challenges and advantages of being a scientific editor; now, we know **how ChatGPT describes us editors**. Disruptive novelties have always caused and will always cause insecurity in people, and this is very good, as it creates the need to observe and reflect on their applications. In the beginning, it was thought that Nikola Tesla’s alternating current would set cities on fire, that the steam engine would explode, and that the computer would leave millions unemployed. However, these and other technological advances have provided a safer and more comfortable life for humanity.

## How do we editors see ChatGPT?

We acknowledge some possible future perspectives for ChatGPT and other similar tools. The use of ChatGPT as a medical writing tool could be taken into consideration in the near future. Not every researcher or scientist is necessarily a good writer. Not every researcher has access to professional medical writers. Language is sometimes is also an important barrier. In this context, if all ethical principles and editorial policies are strictly followed, ChatGPT could be a useful tool, under human supervision, to turn researchers’ data, results, and main conclusions into a proper scientific article. Overall, this would ultimately shorten the time between data analysis and publication of scientific knowledge. It will not be a surprise if GPT can reach an elevated level of compiling data, transcending actual ways of human thinking and creativity, and culminating in scientific content creation.

Employing artificial intelligence (AI) technologies, such as ChatGPT, may bridge the gap between researchers in developing and developed countries. The fierce competition for publication in high-impact scientific journals is exacerbated by linguistic barriers, challenges in effectively presenting and discussing results, crafting abstracts, selecting keywords, and even devising captivating titles, all of which may contribute to the manuscript rejection. As editors, we anticipate keenly observing whether the coming years will witness a surge in contributions from research groups in developing countries, thereby fostering greater inclusivity in the global scientific discourse.

We recognize its incredible advancements in science and technology; however, due to numerous controversies, the **editorial team has decided to not allow the publication of ChatGPT-authored articles in ABO** after the present editorial until we obtain a better understanding and control of the transparency, ethical, and responsible disclaimer of authorship.

Scientific journals should be the spearheads for the dissemination of innovation, and their editors should ensure the origin of the information that reaches readers and preserve copyrights. Thus, by barring, at this moment, the publication of articles originating from AI, it does not mean it will be permanently banned. Until concerns regarding distinctions between editing and authoring, copyright, ethical deviations, and potential infringement liability are more clearly defined, as recently observed, we will adopt a cautious approach^(3,4)^. Among the concerns is the possibility of creating unrealistic narratives and series, i.e., those that do not correspond to reality, generated by AI algorithms. These generative technologies allow users to generate text, images, and videos from little input information, which can lead to the creation of deceptive and false content. It could be dangerous in medical sciences, where the veracity of information is paramount. In addition, disseminating fake news can have serious consequences on public health.

Anti-AI checking tools must be developed and improved. Just as we have strategies for checking plagiarism in texts, we need mechanisms to verify the authenticity of the algorithm-generated content. This may involve the use of machine-learning techniques to detect patterns and inconsistencies in texts, images, and videos.

Our **“Instructions for Authors”** section will be updated incrementally as the scientific community embraces the prudent use of these emerging technologies. This precaution means that **ABO Editors** need time to observe and reflect on its implications before endorsing it for their readers.
